# Reply to Giansanti et al. The *Accessibility* and the *Digital Divide* in the Apps during the COVID-19. Comment on “Cao et al. The Impact of Using mHealth Apps on Improving Public Health Satisfaction during the COVID-19 Pandemic: A Digital Content Value Chain Perspective. *Healthcare* 2022, *10*, 479”

**DOI:** 10.3390/healthcare10071259

**Published:** 2022-07-06

**Authors:** Junwei Cao, Guihua Zhang, Dong Liu

**Affiliations:** 1 Department of Digital Convergence Business, Yeungnam University, Gyeongsan 38542, Korea; 21850211@yu.ac.kr; 2 Department of Business, Yeungnam University, Gyeongsan 38542, Korea; 3 Department of Global Business, Yeungnam University, Gyeongsan 38542, Korea; bruceliu@yu.ac.kr

Thank you for your suggestions for our article [1]. I think it would be very interesting to analyze the role of mHealth apps in COVID-19 from the perspective of the “digital divide” as you suggested, which is something we have ignored in our study. 

We saw in a WHO report the need to prevent the harmful misuse of mobile phone data when countries use such data for epidemic surveillance, and the need to work towards equitable access to cell phones and the Internet for poor people and low-income countries, by investing in infrastructure and designing statistical systems for data collection [2]. We strongly agree with the research suggesting that the digital divide is highly dependent on age [3]. For example, in China’s epidemic prevention and control, the government tracks people’s trips and test results are released through an app on a smartphone, resulting in many older people not being able to travel properly and making it difficult to keep statistics on this demographic. This increasingly highlights the problem of the digital divide.

In terms of mHealth apps, we believe that addressing app accessibility can help alleviate the digital divide, but it is not the main issue. Users of mHealth apps have high demands on aesthetics and ease of use. As such, mHealth apps need to have efficient, intuitive, and easy-to-use application layouts [4,5]. However, there is a lack of research to analyze the design of mHealth apps around the usage characteristics of elderly and disabled people, and there are very few such mHealth apps, which also leads to neglecting the needs of elderly and disabled people when using mHealth apps on a large scale in epidemics. Fortunately, however, electronic device suppliers have made a lot of effort in helping people with disabilities to use their devices. For example, current smartphones, whether IOS or Android, have built-in intelligent assistants that can help users operate most of the functions of the app on the device by voice. This to some extent alleviates the problem of the digital divide between the elderly and the disabled.

The accessibility of the electronic device that hosts the mHealth app is more important than the accessibility of the mHealth app. The study notes that citizens in this epidemic are often unable to use mHealth due to lack of access to tools, cultural barriers, communication barriers, and social barriers [3]. Therefore, we should address the social issues that hinder the accessibility of electronic devices, by reducing the price of electronic devices and increasing education on the use of electronic devices for the elderly and people with disabilities.

The core of this response focuses on another aspect of the secondary digital divide created by too much information and overly complex system provisions. That is, with the attention of society, the elderly and other people with disabilities are already able to use digital systems easily, but as the market demand increases, the continuous complexity of the functions of these digital systems makes it more and more difficult for people with disabilities to use them (creating a second digital divide) and eventually they stop using them. Therefore, I think that with the developments of the times, the digital divide for the elderly and the disabled may not necessarily appear in the accessibility of software and hardware, but instead will appear after the use of the app. With the development of information technology, apps give more and more information to users and information becomes more and more accessible, which leads to the phenomenon of information overload. Information overload is closely related to age and knowledge reserve [6,7], information overload can lead to user fatigue, fear, and other psychological problems, eventually leading them to stop using the app [8]. Specifically, an elderly person could have used the mobile medical app normally, but the large amount of information about the epidemic in a short period of time could have overwhelmed the elderly person, which caused information overload. At the same time, with the development of the epidemic, the mobile health app was updated with a large number of new features in a short period of time, and the elderly could not understand these features in a short period of time, which caused system feature overload, and eventually, the elderly stopped using it or even resisted using it. Information overload may also trigger an information cocoon effect, keeping seniors forever stuck in homogenized information and unable to receive new information. However, the relationship between information overload and the digital divide is not yet fully confirmed, so the digital divide caused by inappropriate use is also well worth studying. In COVID-19, such a phenomenon is obvious, the Chinese government promotes several QR codes in epidemic prevention, people need to show these QR codes to epidemic prevention officials frequently when they travel, these QR code generation functions are integrated in some apps that are already very popular, many elderly people will use these apps but cannot find the location of these QR codes in the apps, and this creates a digital divide due to too much information (i.e. too many new features affecting the use, too much information becomes difficult to search). There are already companies in China that have made improvements for such problems, for example, some companies have developed a button that can be easily attached to the back of smartphones, and when the elderly need to display these QR codes, they can reach the function by pressing the button, which is convenient for the elderly population, but the intention of using this tool and the market prospect are still not clear enough and need further research.

We have several ideas for the follow-up study. First, we can design a model based on the updated information system success theory to introduce some variables related to app accessibility and the accessibility of electronic devices hosting apps, and improve the current mHealth app operation problem and electronic device penetration problem to reduce the occurrence of digital divide in elderly years by demonstrating the effects of these variables on the satisfaction of elderly people’s usage and the degree of usage. Secondly, I think an experiment can be designed to give different amounts of information stimuli to compare the characteristics of users using mHealth apps under different information overloads, and based on the results, suggestions can be made to design the operation of mHealth apps in the epidemic to reduce the digital divide caused by inappropriate use. Thirdly, policy factors and family factors are also variables worth including in the analysis to help address the impact of the digital divide on the use of mHealth apps from a social perspective. Finally, a model can be designed to analyze the usage intentions and market prospects of “one-touch” devices that currently help older adults address the second digital divide.

In conclusion, thank you again for your comment on our research. We will learn from your research in the area of digital divide and follow your suggestions for our next research study.
